# Supplementary material for: Structural Characterization of a Minimal Antibody against Human APOBEC3B
Source: Viruses. 2021 Apr 12;13(4):663. doi: 10.3390/v13040663 (PMC8070380; doi:10.3390/v13040663)

Supplementary Fig. 1. Root-mean-square deviation of 5G7 Cα atoms in MD simulations with respect to the initial binding pose. The superimposition is performed according to 5G7 Cα atoms.

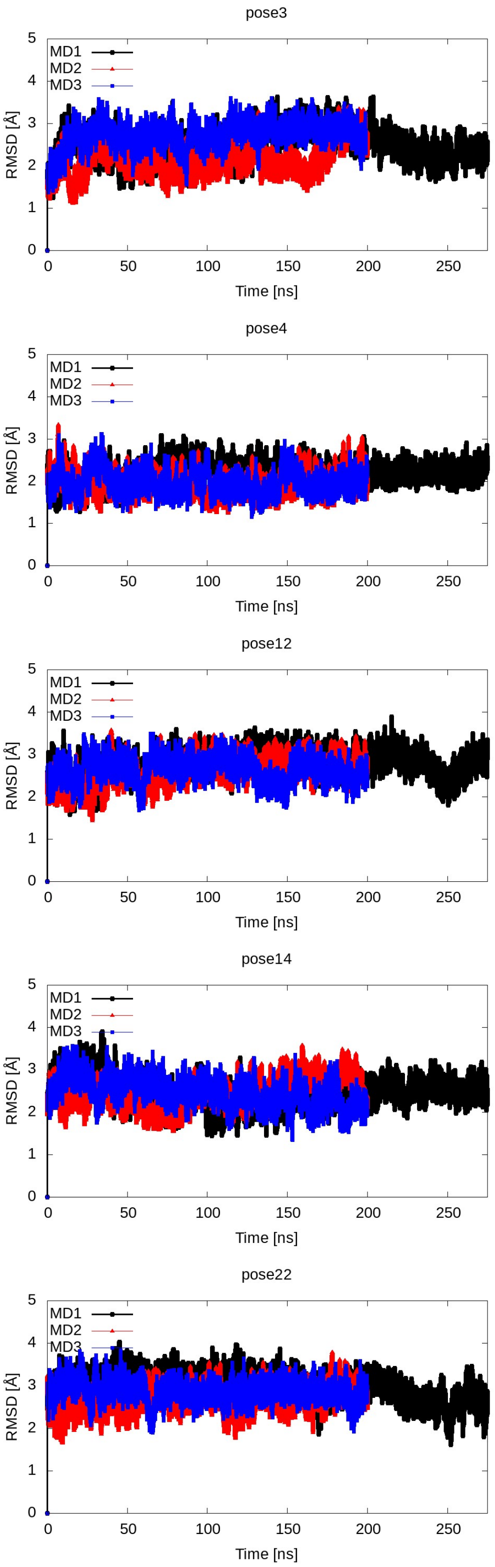

Supplementary Fig. 2. Root-mean-square deviation of A3B Cα atoms in MD simulations with respect to the initial binding pose. The superimposition is performed according to 5G7 Cα atoms.

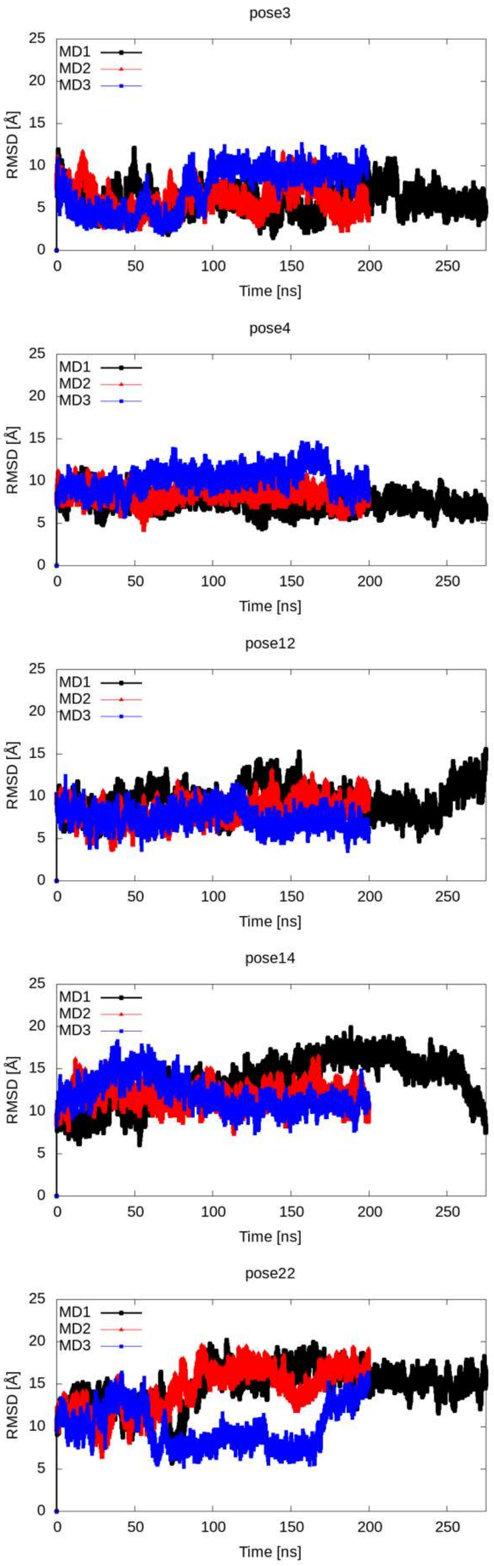

Supplementary Fig. 3. Root-mean-square deviation of A3Bctd α6 helix Cα atoms in MD simulations with respect to the initial binding pose. The superimposition is performed according to 5G7 Cα atoms.

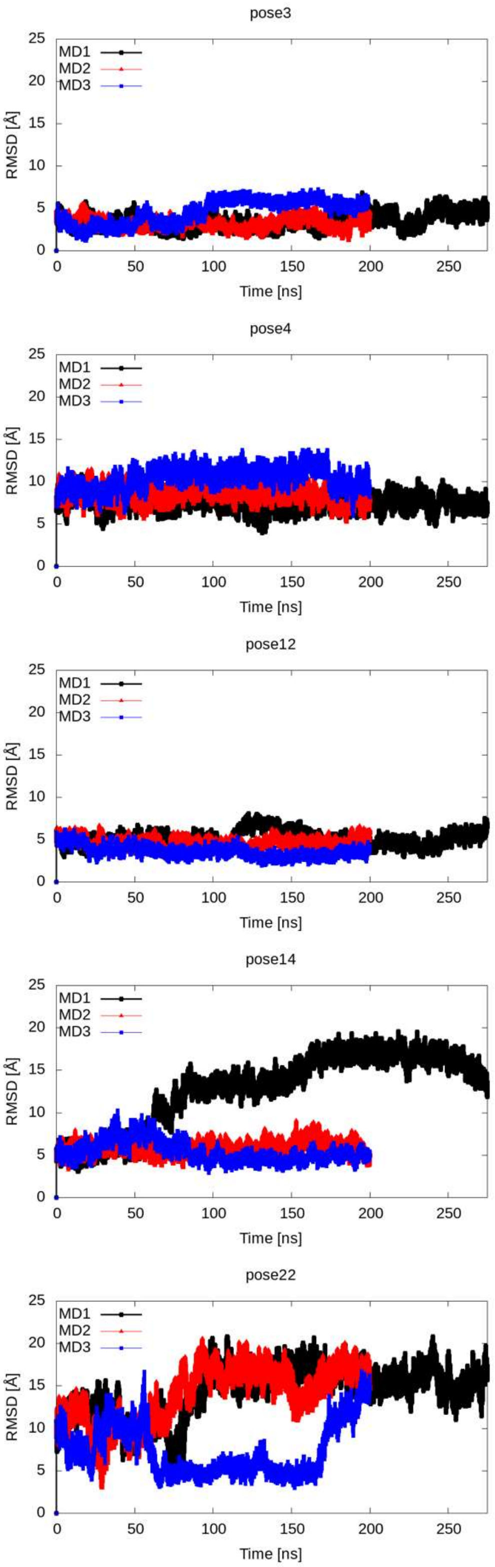

Supplementary Fig. 4. Distance between A3Bctd Arg374 Cα atom and Cα atom of its interaction partner in 5G7 in MD simulations.

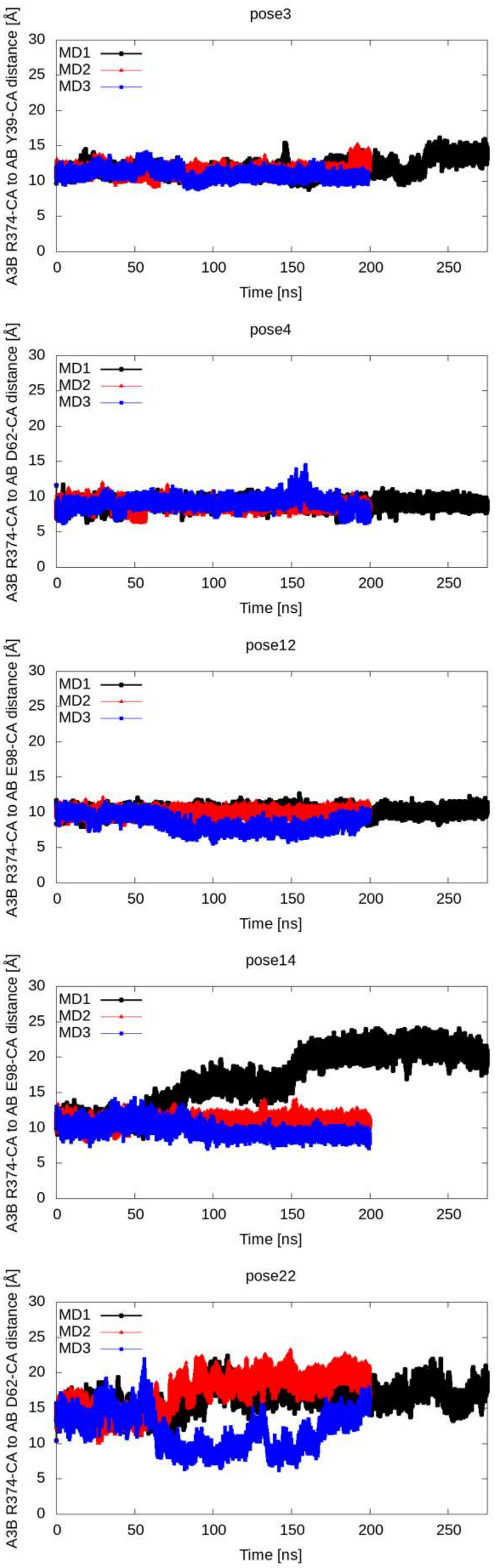

Supplementary Fig. 5. Distance between A3Bctd Arg374 CZ atom and the heavy (non-hydrogen) atom at the tip of its interaction partner sidechain in 5G7 in MD simulations.

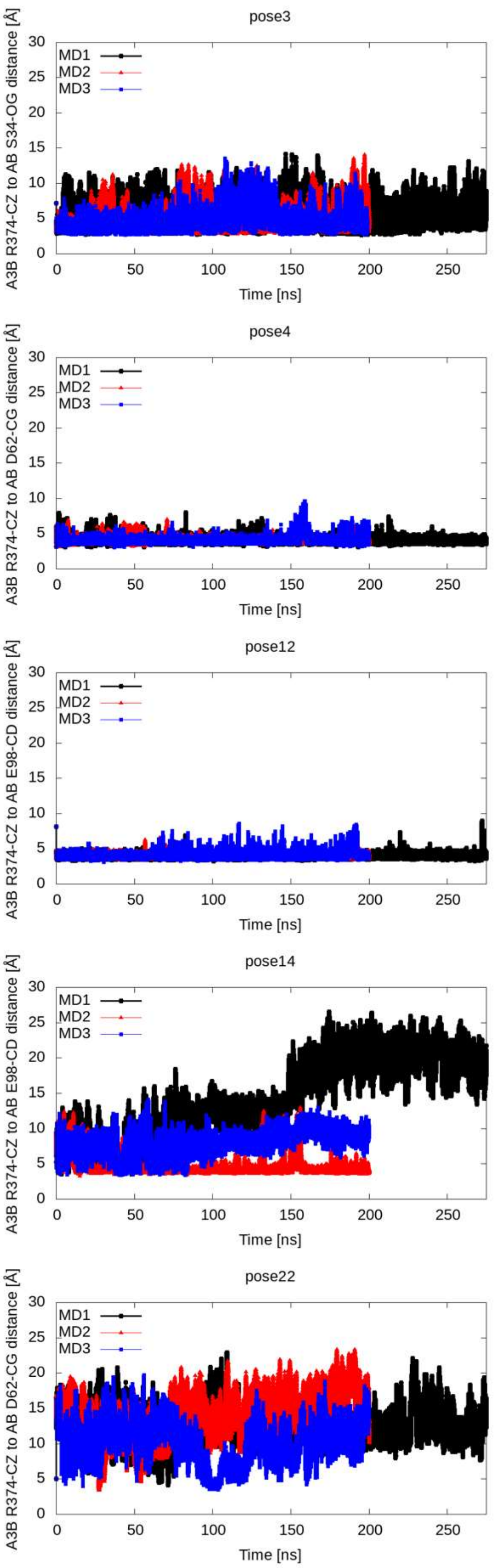

Supplementary Fig. 6. Mapping of A3Bctd vs. A3A (left) or A3Bctd vs. A3Gctd (right) amino acid sequence divergence on the proposed structure (binding pose #3) of the 5G7-A3Bctd complex. Non-identical residues are highlighted by color with side chains shown as sticks. Ala368 of A3Bctd is conserved in A3A but it corresponds to Asp370 of A3G, which may account for the cross-reactivity of 5G7 with A3A but not with A3Gctd.

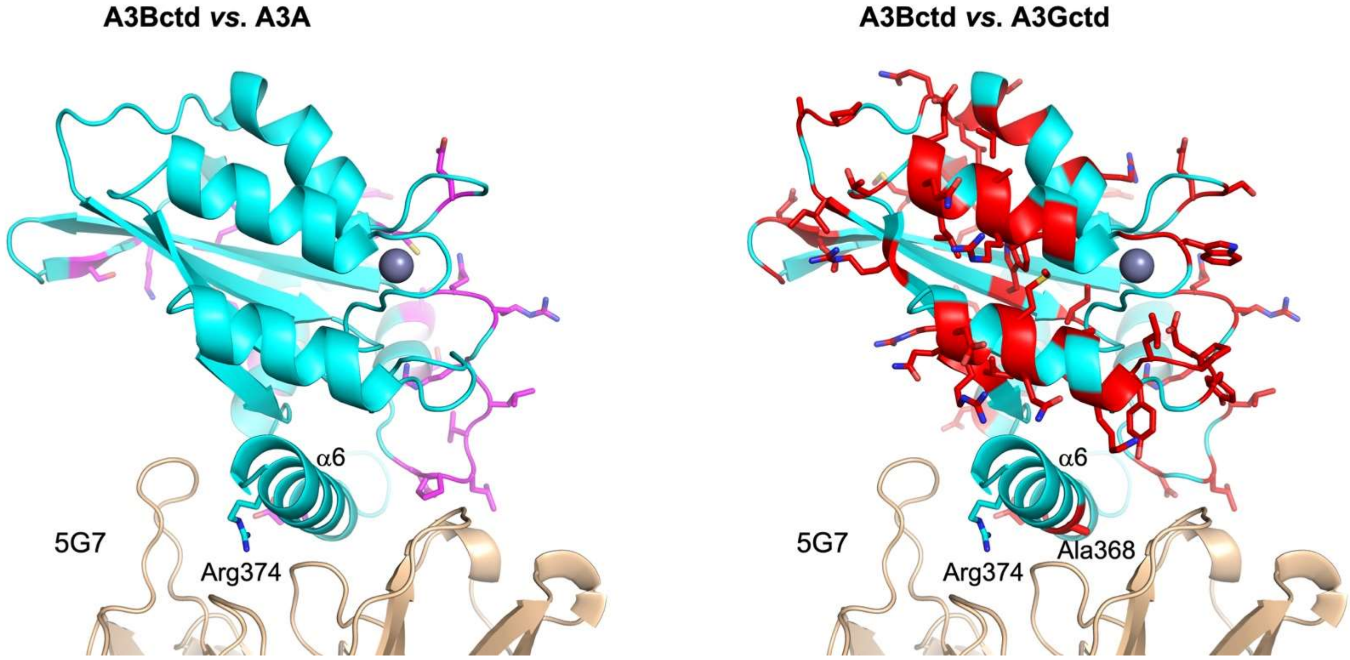

Supplement: Supplementary file 1 [file viruses-13-00663-s001.pdf]
